# Regulatory activity based risk model identifies survival of stage II and III colorectal carcinoma

**DOI:** 10.18632/oncotarget.21312

**Published:** 2017-09-28

**Authors:** Gang Liu, Chuanpeng Dong, Xing Wang, Guojun Hou, Yu Zheng, Huilin Xu, Xiaohui Zhan, Lei Liu

**Affiliations:** ^1^ Shanghai Public Health Clinical Center and Institutes of Biomedical Sciences, Fudan University, Shanghai, China; ^2^ The Third Department of Hepatic Surgery, Eastern Hepatobiliary Surgery Hospital, Second Military Medical University, Shanghai, China; ^3^ CAS-MPG Partner Institute for Computational Biology, Shanghai Institutes for Biological Sciences, Chinese Academy of Sciences, Shanghai, China

**Keywords:** colorectal cancer, prognosis, transcription factor, model

## Abstract

Clinical and pathological indicators are inadequate for prognosis of stage II and III colorectal carcinoma (CRC). In this study, we utilized the activity of regulatory factors, univariate Cox regression and random forest for variable selection and developed a multivariate Cox model to predict the overall survival of Stage II/III colorectal carcinoma in GSE39582 datasets (469 samples). Patients in low-risk group showed a significant longer overall survival and recurrence-free survival time than those in high-risk group. This finding was further validated in five other independent datasets (GSE14333, GSE17536, GSE17537, GSE33113, and GSE37892). Besides, associations between clinicopathological information and risk score were analyzed. A nomogram including risk score was plotted to facilitate the utilization of risk score. The risk score model is also demonstrated to be effective on predicting both overall and recurrence-free survival of chemotherapy received patients. After performing Gene Set Enrichment Analysis (GSEA) between high and low risk groups, we found that several cell-cell interaction KEGG pathways were identified. Funnel plot results showed that there was no publication bias in these datasets. In summary, by utilizing the regulatory activity in stage II and III colorectal carcinoma, the risk score successfully predicts the survival of 1021 stage II/III CRC patients in six independent datasets.

## INTRODUCTION

Colorectal carcinoma (CRC) is one of the most important causes of death worldwide [1]. According to recent reports, 376,300 new cases and 191,000 deaths occurred due to CRC, in China, 2015 [2]. Currently, the prognosis of colorectal cancer is controversial in stage II and III colorectal carcinoma [3]. Although the staging system is mature, some stage II colorectal adenocarcinoma patients have relatively poorer prognosis than stage III CRC patients. This indicates that clinical observations, including stage, could not distinguish the good or poor prognosis of colorectal carcinoma well in stage II/III CRC.

During the past years, numerous molecular biomarkers have been reported to be able to predict the survival of stage II and III colorectal carcinoma patients [4–8]. However, the single biomarker's prognostic value is usually unfavorable across datasets. In order to elevate the performance on prognosis, multiple gene models for predicting survival of carcinomas have been developed [9–12]. The expression of genes, especially cancer-related genes, are regulated by critical signaling pathways and transcription factors [13–15], The transcription factor activity of core signaling pathways reflects the cell status and cancer heterogeneity.

In this article, we evaluated the activities of regulatory factors, and then developed a Cox multivariate model to predict the survival of stage II and III colorectal carcinoma patients from GSE39582 dataset. The risk score is significantly associated with overall and recurrence-free survival. The performance of risk score model in predicting survival of stage II and III colorectal adenocarcinoma was further validated in five independent datasets. Association analysis showed that the risk score was independent from clinical information including age, stage and gender. A nomogram was plotted to facilitate the utilization of risk score. In conclusion, transcription regulation activity based risk score successfully predict the survival of stage II and III colorectal carcinoma.

## RESULTS

### Candidate gene selection and model development

Detailed information of datasets used in this study were listed in Table 1. Regulators including transcription factors and core pathway genes were important for cancer development. However, the activity of these regulators could not be assessed by the mRNA level because some regulators took effect by protein modifications, thus, the regulatory activity of regulators was calculated based on the expression levels of target genes downstream. The survival significance of candidate regulators (based on their regulatory activity) was evaluated using cox univariate regression (p<0.05). Forty-four regulator activities were detected to be correlated with survival, then random forest was implemented for variable hunting. Totally, ten regulators’ activities (EPAS1, TP73, TEAD1, DBP, NME2, GFI1, NR5A1, ELK1, NANOG and ETS2) were selected as candidate features (regulators). Cox multivariate analysis was performed with above candidate regulators, and the coefficients of each regulator were assigned as its weighting, respectively (Table 2). The hazard ratios <1 suggested that their corresponding regulators were tumor suppressor genes, while genes with hazard ratios >1 were cancerous genes.

**Table 1:** Sample size and survival information of datasets used in this article

Datasets	Sampls	Survival info provided
GSE143	162	Disease-free survival
GSE176	115	Overall survival
GSE177	55	Overall survival
GSE333	90	Progression-free survival
GSE272	130	Metastasis-fee survival
GSE396	469	Overall survival

**Table 2:** Basic parameters of regulatory factors used for risk score

TFs	Genes downstream	Coefficiens	Frequeny	Hzazad ratio	CI 95%	p Value
DBP	1	1.4199301	25	1.39	1.04-1.85	0.026
ELK1	3	0.1978697	28	1.77	1.21-2.59	0.0036
EPAS1	3	0.219671	24	1.59	1.09-2.31	0.015
ETS2	2	0.3803902	34	1.93	1.16-3.22	0.011
GFI1	5	-0.237219	26	0.523	0.331-0.829	0.0057
NANOG	2	-3.168149	33	0.642	0.415-0.993	0.046
NME2	5	-0.130288	26	0.736	0.607-0.893	0.0018
NR5A1	2	0.0413388	28	3.83	1.09-13.5	0.037
TEAD1	1	0.5805332	25	1.22	1.1-1.35	0.000091
TP73	383	-0.166284	25	34.9	1.33-920	0.032

The columns are number of genes used for regulatory factor evaluation, Cox univariate regression p value, Cox multivariate regression beta values, and frequencies of regulatory factors of random forest variable hunting.

### Risk score predicts survival in the training dataset

**Figure 1:**
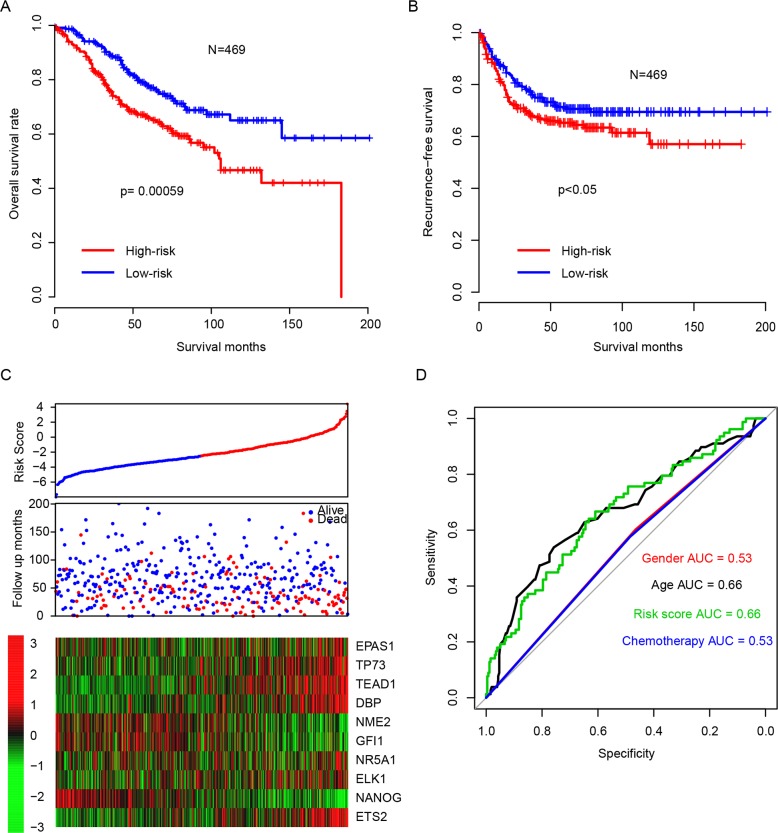
Performance of regulatory factor activity based risk score The high-risk group has a significant longer overall survival **(A)** and recurrence-free survival **(B)** time than low-risk group. The detailed survival information and regulatory factor activity **(C)** and three-year survival ROC **(D)** was shown.

**Figure 2:**
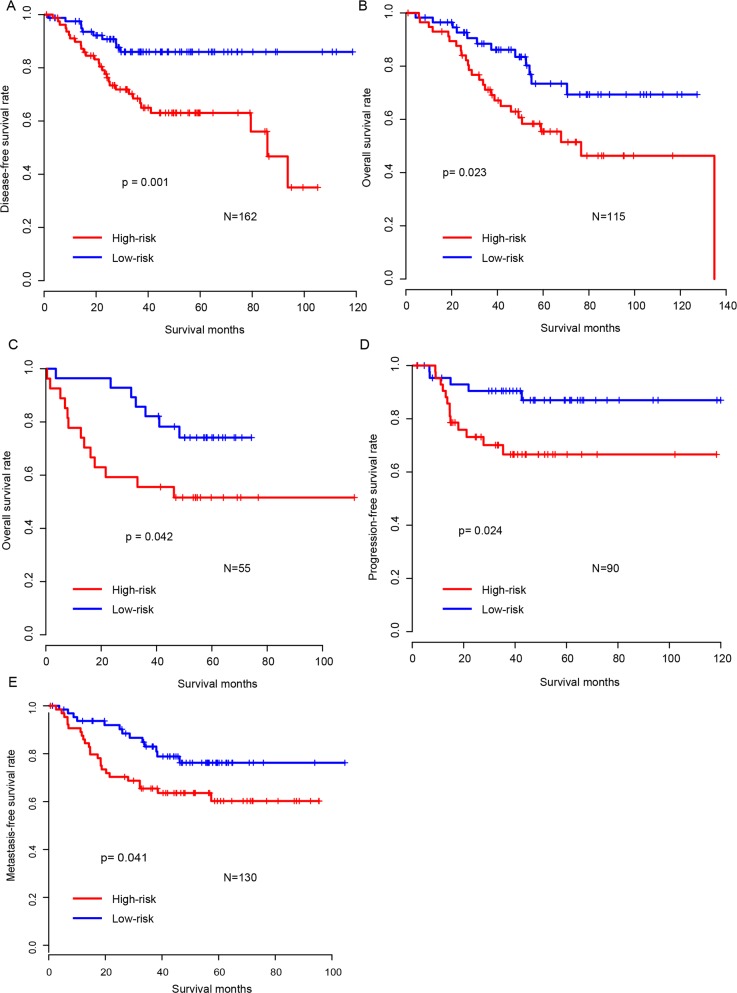
Validation of survival-predicting performance of risk score The performance of risk score was further validated in five independent datasets (**A**: GSE14333, **B**: GSE17536, **C**: GSE17537, **D**: GSE33113, **E**: GSE37892).

After developing risk score staging model in the training dataset, the survival-predicting value of risk score was evaluated. The patients were subtyped into high risk (n = 235) and low risk (n = 234) group by using the median risk score value as cutoff. The overall survival (OS) of high-risk group was significantly shorter than the low-risk group (Figure 1A, p=0.00059). In addition, the recurrence-free survival (RFS) profile of high-risk group resembled that of its overall survival (Figure 1B, p<0.05). Detailed survival information and risk scores were shown in Figure 1C. The regulatory activity pattern of the candidate genes was consistent with their coefficients. The risk score performs better in predicting the three-year survival of stage II and III CRC patients compared with clinicopathological indicators (Figure 2D). Area under receiving operating characteristic curve (AUROC) for three-year survival was plotted, showing a result of 0.66 for risk score and 0.66, 0.53, 0.53 for age, gender, chemotherapy, respectively. This indicated that the risk score was an important survival indicator for stage II and III colorectal carcinoma.

### Risk score model is robust across the test datasets

The high performance of risk score model in the training dataset may result from over-fitting. To assess its robustness, we carried out risk score performance evaluation on five independent public CRC cohorts, after locking the coefficients of the model. It was shown that the survival time of patients in the high-risk group was significantly shorter than in that the low-risk group, which was in consistent with the survival profile of training datasets (Figure 2A-E). In addition, the regulatory activity of candidate genes in the test datasets also resembled that in training datasets (Supplementary Figure 1A-E). These results above indicate that our risk score model was robust across datasets.

### Risk score and clinical/pathological indicators

**Figure 3:**
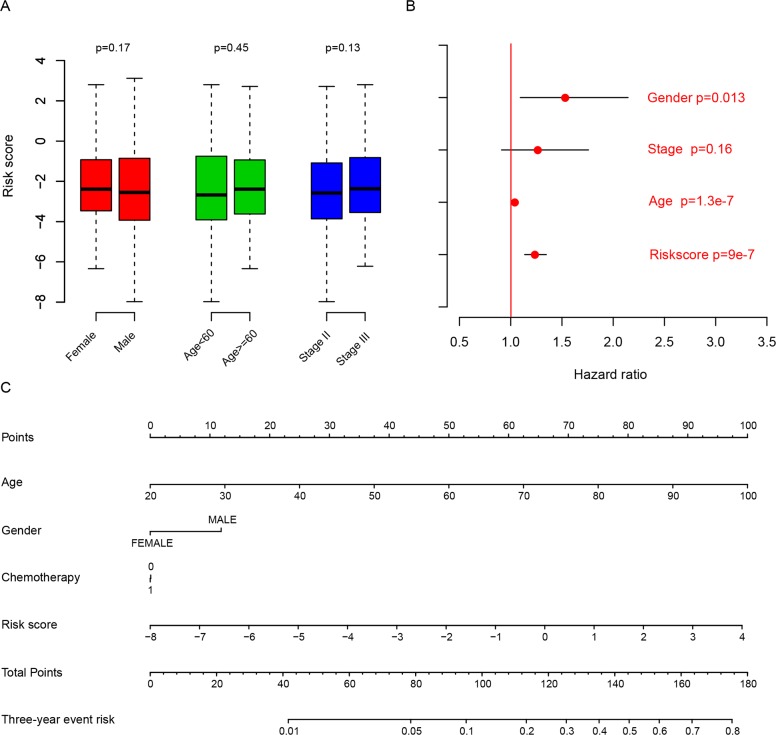
Risk score and another clinical indicator The risk score is independent from age, gender, and stage **(A)**, and is an important clinical indicator for survival according to multivariate hazard analysis **(B)** and nomogram **(C)**.

The relationship between the risk score we developed and clinical/pathological information was measured as well (Figure 3A). It was found to be independent from gender, age and stage (p>0.05). Multiple cox hazard ratio analysis results showed that risk score was an important indicator for predicting survival (Figure 3B). In order to facilitate the utilization of risk score model, a nomogram including gender, age, stage, risk score and chemotherapy was plotted (Figure 3C). The Cox univariate and multivariate regression of risk score and more detailed information indicated that risk score was the most important indicator for prognosis, as shown in Table 3. These results indicated that risk score was an independent and critical indicator for prognosis.

**Table 3:** Cox univariate and multivariate regression of clinical indicators in GSE39582

Gene	Univariate regression			Multivariate regression		
	HR	95%CI	p Value	HR	95%CI	p Value
Riskscore	1.2	1.1-1.3	0	1.15	1.04-1.27	0.00692
SexM	1.3	0.91-1.8	0.16885	1.35	0.91-2	0.13668
Stage	1.2	0.89-1.7	0.20707	1.17	0.79-1.73	0.44657
Location	1.1	0.81-1.6	0.472	1.04	0.67-1.6	0.86886
CIMP	0.93	0.57-1.5	0.76494	0.73	0.34-1.55	0.41108
CIN	0.99	0.62-1.6	0.9745	0.93	0.54-1.58	0.78014
KRASmut	1.4	1-2	0.03751	1.47	0.97-2.24	0.07001
BRAFmut	0.89	0.46-1.7	0.71906	1.35	0.52-3.5	0.53881
CDX2	0.83	0.7-0.98	0.02838	0.92	0.7-1.19	0.50933

### Risk score and chemotherapy

**Figure 4:**
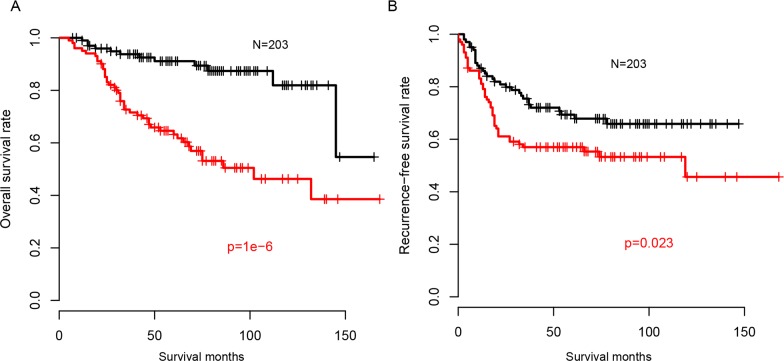
Risk score and chemotherapy Overall survival **(A)** and recurrence-free survival **(B)** of patients underwent chemotherapy in high risk group is longer than in low risk group.

Chemotherapy is the one of most important adjuvant treatment strategies following surgery. Thus, the correlation between risk score and chemotherapy was evaluated. We used overall survival and recurrence-free survival information to estimate the availability of our risk score model for predicting the survival of patients with chemotherapy. As expected, the chemotherapy received patients with high risk score had a worse prognosis both on overall survival (Figure 4A) and recurrence-free survival (Figure 4B), compared to the low risk group. The prognostic value of risk score was also evaluated in patients without chemotherapy, and it was similar with chemotherapy-receiving group (Not shown). These results indicated that the regulatory activity based risk score was also available for the prognosis of CRC patients with chemotherapy.

### Identification of biological pathways associated with risk score

**Figure 5:**
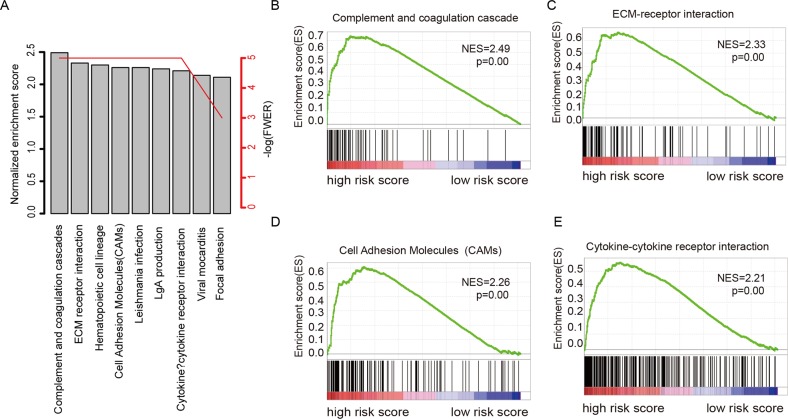
KEGG pathways associated with risk score Of the KEGG pathways significantly associated with risks score **(A)**, complements and coagulation cascades **(B)**, ECM receptor interaction **(C)**, cell adhesion molecular cams **(D)**, and “cytokine-cytokine receptor interaction” were noted.

In order to investigate why the risk score can predict the survival of colorectal carcinoma, the comparison of gene expression profile between high-risk and low-risk group was performed, according to the median value of risk score in the largest cohort, GSE39582. The altered KEGG pathways was evaluated using Gene Set Enrichment Analysis (Figure 5A). The results showed that the most altered and enriched KEGG pathways were “complements and coagulation cascades” (Figure 5B), “ECM receptor interaction” (Figure 5C), “cell adhesion molecular” (Figure 5D), and “Cytokine-cytokine receptor interaction” (Figure 5E). These results indicated a possible molecular mechanism of the clinical outcome in stage II and III colorectal adenocarcinoma reflected by risk model.

### Publication bias evaluation

**Figure 6:**
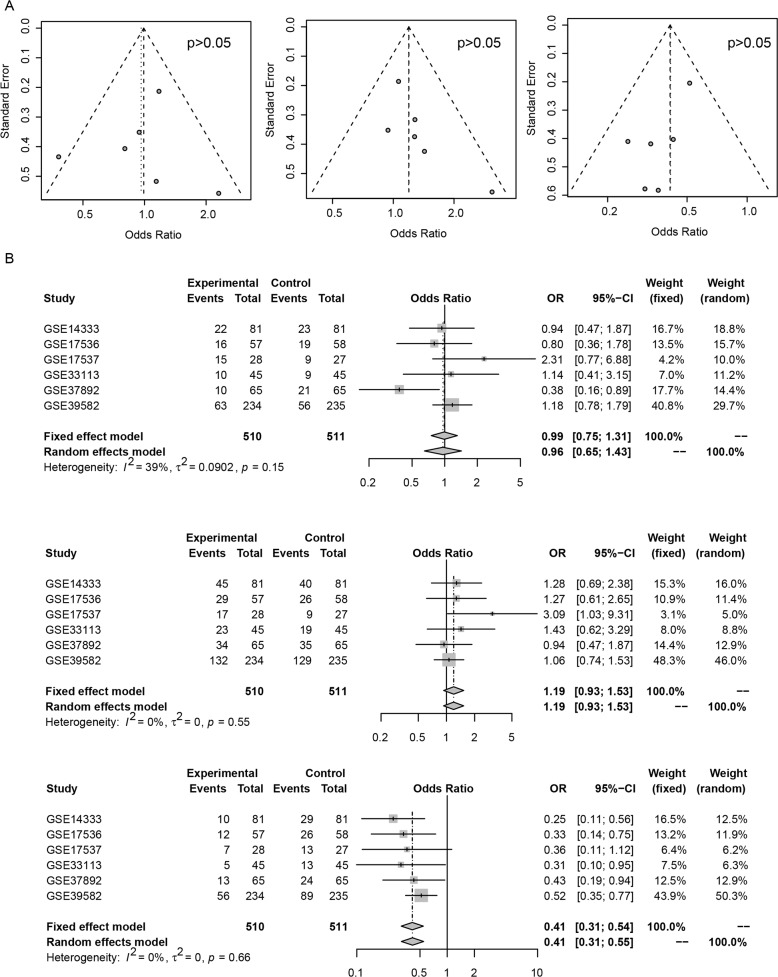
Publication bias of risk score and clinical indicator Funnel plot of age (**A**, left), gender (A, middle), and events (A, right) has no bias. Forest plot suggests the similar results (**B**, top-down, age, gender, events).

Publication bias inspection regarding basic clinical information, including age, gender, and events (relapse, metastasis, death) was performed. Funnel plots indicated that no publication bias for gender, age, or events was detected (Figure 6A, p>0.05). The forest plot showed that no data heterogeneity exists (Figure 6B). Publication bias was not investigated when the number of studies was less than 10 because of the low sensitivity of the qualitative and quantitative tests [16].

## DISCUSSION

Prognosis of stage II and III colorectal carcinoma still remains a problem. Although single biomarker has been reported for survival prediction [8, 17, 18], the robustness of these biomarkers still remains a huge concern. One of the reasons may be that single biomarker fails to reflect the genomic heterogeneity of tumors. Regulatory factors control the expression of genes downstream, and further determine the status of crucial pathways. Activity of multiple core regulatory and transcription factors may reflect the genomic status of cancer cells. In this vein, we evaluated the activities of transcription and regulatory factors by considering the expression of target genes downstream of stage II and III colorectal carcinoma. Using cox univariate regression and random forest variable hunting, activities of ten regulatory factors were identified to develop a risk score model for prognosis. The model successfully predicted survival of 1021 stage II and III colorectal carcinoma patients in six independent datasets. It is also independent from other clinical indicators and performs exceedingly in survival-predicting.

We noticed that the most of the 42 regulators are important for prognosis, the combination of the ten regulators effectively reduced the panel and retained the useful information. Among the ten transcription regulators, we noted that the overexpression of EPAS1 was associated with poor prognosis in colorectal carcinoma, according to previous reports [19–21]. Polymorphism and expression of TP73 were associated with carcinogenesis and colorectal carcinoma development [22, 23]. TEAD1 was reported to enhance the proliferation in colorectal carcinoma [24]. DBP and NME2 were associated with carcinogenesis and development of cancer types, including colorectal carcinoma [25–28]. It was similar for GFI1 [29–31], NR5A1 [32], and ELK1 [33–35]. NANOG was related to multiple colorectal tumor development functions, including liver metastasis [36], stemness maintaining [37] and prognosis [38]. ETS2 was shown to be associated with metastasis of colorectal carcinoma [39, 40]. These reports indicated that the regulatory factors included in the risk score model were essential prognostic genes, implying the reliability of this model.

The metastasis of CRC is among the most serious events during colorectal carcinoma development [41]. Among pathways and genes involved in CRC metastasis, cell-cell focal adhesion plays important roles [42, 43]. According to GSEA analysis, the most pathways involved in cell-cell interaction and focal adhesion were significantly enriched, which may explain why risk score is associated with stage II/III CRC prognosis.

In conclusion, our transcription activity based risk score model successfully predicts the survival of stage II and III colorectal carcinoma. To our knowledge, this is the first model using activities regulatory factors to predict survival of stage II/III colorectal carcinoma.

## MATERIALS AND METHODS

### Data preprocessing

The raw data of six datasets (GSE39582, GSE14333, GSE17536, GSE17537, GSE33113 and GSE37892) was downloaded in. CEL format. After background correction and normalization, the fold change between expression value of each sample and median expression value for each gene was calculated. Probes were matched to the gene names, and genes matching more than one probe were merged and average values were calculated as the expression of the corresponding genes. Duplicated values were excluded. The regulatory factor-downstream pairs were constructed according to the regulatory data provided by HTRI database [44]. Suppose the downstream genes of regulator k (R_k_) are Gene_1,2,3…j_, and the dataset consist of samples 1,2,3…i.

**Table:** 

Sample 1		sample 2		sample 3		…	sample i
Gene 1	…	…	…	…	…	…	
Gene 2	…	…	…	…	…	…	
Gene 3	…	…	…	…	…	…	
…	…	…	…	…	…	…	
Gene j	…	…	…	…	…	…	

The regulator factor activity (RFA) of regulator k is calculated as the following,

$${\text{RFA}}_{k,i}=\sum_{i}^{n}\left[{\text{Gene}}_{j,i}-\text{median}({\text{Gene}}_{j})\right]$$

Where Gene_j,i_ indicates the gene expression value of Gene_j_ in sample i, and median (Gene_j_) refers to the median expression values of Gene_j_, refers to the regulatory factor activity of regulator k in sample i. Construct a new matrix containing activity of regulators, in which the rows represent the regulators and the columns indicate the samples. All datasets included in this article was transformed using the same method.

### Gene selection and model construction

Cox univariate regression was performed on GSE39582 dataset. Transcription factors that significantly associated with overall survival in this dataset were retained. Random forest variable hunting was performed with 100 replications and 100 steps. Multivariate Cox regression was implemented to construct the risk score model with the candidate genes, and coefficients were locked in the five test datasets. The risk scores (RS) of each sample were calculated as the following,

$$RS_{i}=\sum_{k}^{n}{\text{RFA}}_{k,i}*\beta_{k}$$

Where indicates the regulatory factor activity of regulator k in sample i, and β_i_ refers to the coefficients for candidate regulators. Coefficients was evaluated using the training dataset, GSE39582, and locked to calculate the risk score in the other five datasets (GSE14333, GSE17536, GSE17537, GSE33113 and GSE37892). The median risk score values in each dataset were used as cutoff to identify the high-risk and low-risk group.

### Statistical analyses

All statistical analysis was performed on R language and R packages. Microarray data pre-process was performed with R package “affy”. Survival analysis, Cox univariate regression and Cox multivariate regression were carried out with R package “survival”[45], and random forest variable hunting was implemented with R package “randomForestSRC”[46]. Survival ROC curve was plotted with R package “pROC”[47], and nomogram was drawn with R package “rms”[48]. Publication bias analysis was performed on R package “meta”. Gene Set Enrichment Analysis was carried out on java software “GSEA”[49].

## SUPPLEMENTARY MATERIALS FIGURE


