# Supplementary material for: Regulatory activity based risk model identifies survival of stage II and III colorectal carcinoma
Source: Oncotarget. 2017 Sep 28;8(58):98360–70. doi: 10.18632/oncotarget.21312 (PMC5716735; doi:10.18632/oncotarget.21312)
Supplement: Supplementary file 1 [file oncotarget-08-98360-s001.pdf]

Regulatory activity based risk model identifies survival of stage II and III colorectal carcinoma

SUPPLEMENTARY MATERIALS

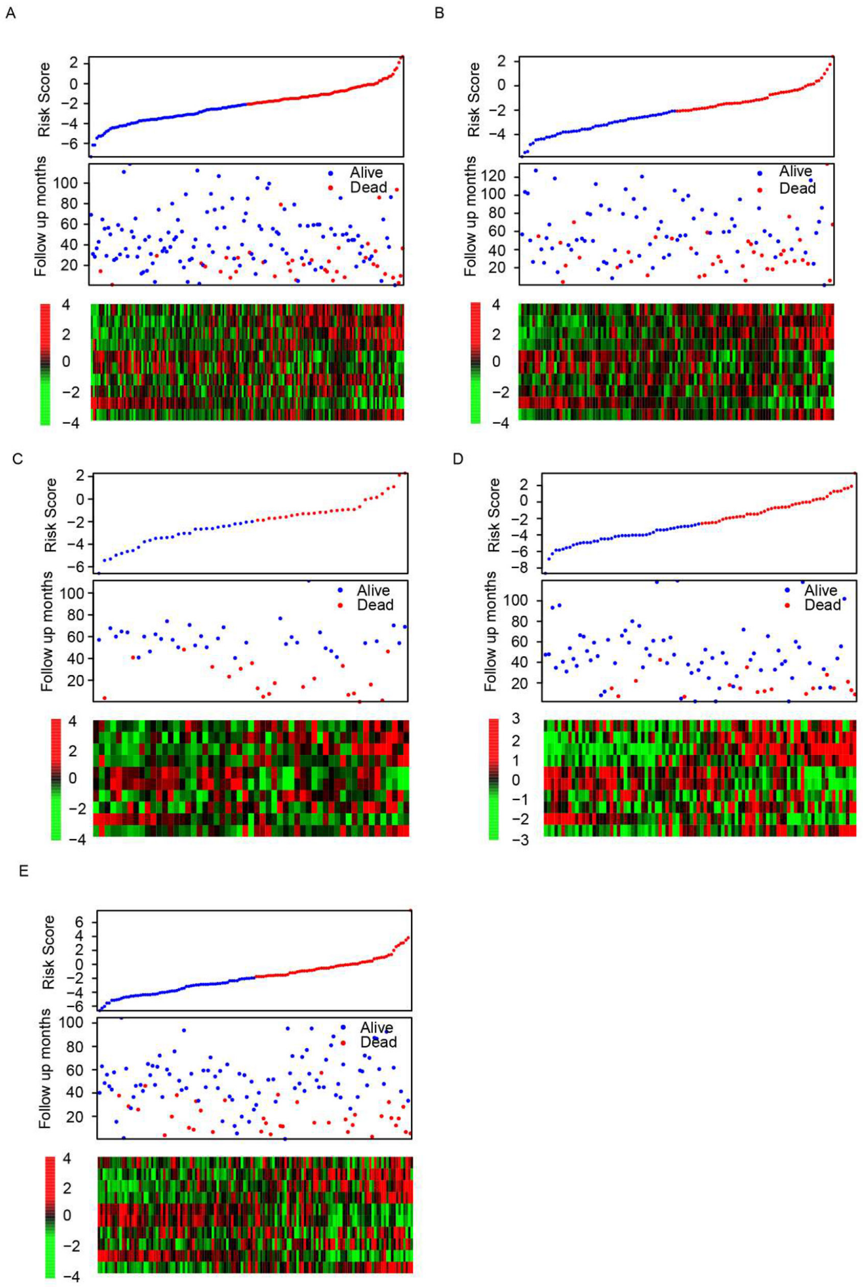

Supplementary Figure 1: The detailed survival information and transcription activity of candidates in five independent datasets **(A)** GSE14333, **(B)** GSE17536, **(C)** GSE17537, **(D)** GSE33113, **(E)** GSE37892).
